# Implementation of the World Health Organization Global Burn Registry: Lessons Learned

**DOI:** 10.5334/aogh.3669

**Published:** 2022-05-18

**Authors:** Caitlin Hebron, Kajal Mehta, Barclay Stewart, Patricia Price, Tom Potokar

**Affiliations:** 1 Centre for Global Burn Injury Policy and Research, Swansea University, Wales, UK; 2 Interburns, Swansea, UK; 3 Department of Surgery, University of Washington, Seattle, WA, USA; 4 Division of Trauma, Burn, and Critical Care Surgery, Department of Surgery, University of Washington; 5 UW Medicine Regional Burn Center, Seattle, WA; 6 Harborview Injury Prevention & Research Center, Seattle, WA, USA; 7 Interburns, Cardiff, UK

## Abstract

Burn injuries are a major cause of death and disability globally; however, the true epidemiologic burden is underestimated given the limited and fragmented availability of high-quality burn injury data from many regions. To address this gap, the World Health Organization (WHO) Global Burn Registry (GBR)—a minimum dataset aligned with a centralized registry—was officially launched in 2018 to facilitate hospital-level collection of key prevention, care, and outcome data from burn-injured patients around the world in a standardized manner. However, uptake and use of GBR has been low and inconsistent. Therefore, we aimed to identify and understand the barriers and facilitators to the implementation of the GBR to inform the development of a web-based GBR implementation guide through the *Centre for Global Burn Injury Policy and Research* and *Interburns*. We designed and conducted web-based surveys with “GBR users” and “GBR non-users” using purposive sampling. Themes of identified barriers and facilitators focused on awareness of the GBR, stakeholder buy-in, resource constraints, process management, and utility of the registry. The lessons learned could support current and future GBR users to promote and maximize the use of the GBR. To achieve the GBR’s full potential in global burn injury prevention and care, engagement with the GBR should be enhanced through education and promotion, development of a community of practice, tools for data utilization and quality improvement, and periodic re-evaluation.

## Introduction

Burn injuries are a major cause of death and disability globally, with a disproportionate burden incurred by people in low- and middle-income countries (LMICs) [1]. There are nearly 9 million new burn injuries and 120 000 burn injury-related deaths annually [2]. However, these figures likely underestimate the true health burden given the fragmented and limited availability of high-quality burn injury epidemiological data, particularly from LMICs where injury surveillance systems are rare [1]. Without a more accurate estimate of the true burden of burn injuries, it is challenging to adequately target and prioritize injury prevention and control measures.

**Figure 1:**
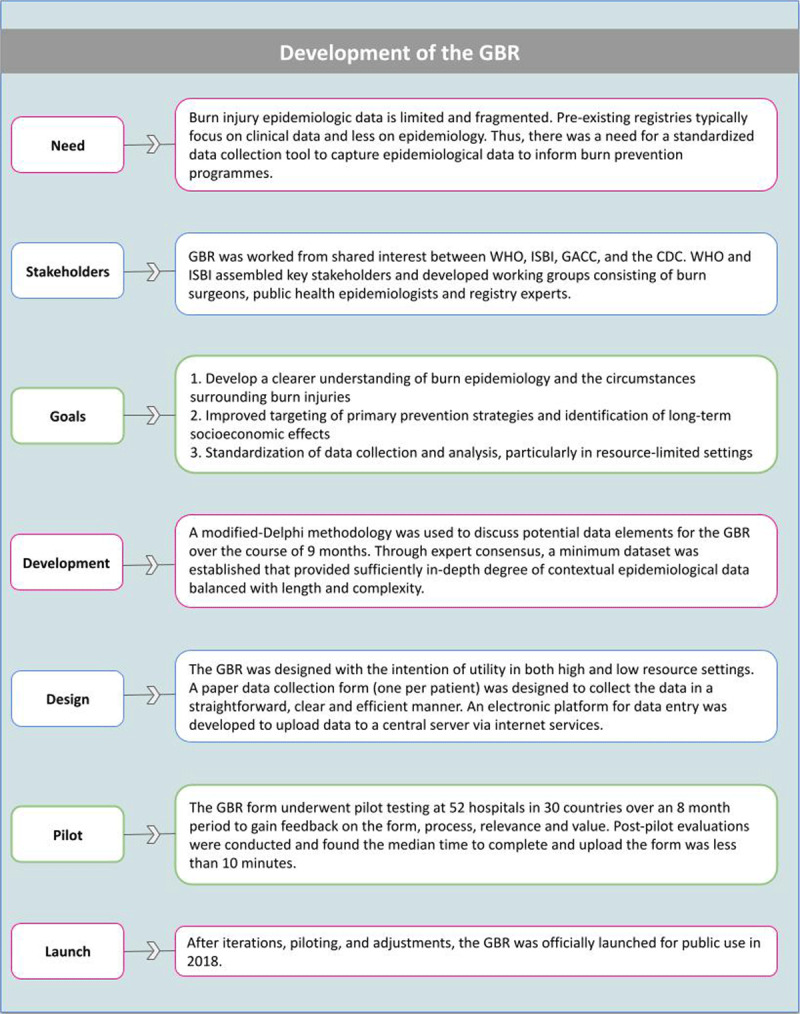
Steps, stakeholders, and development process of the GBR. (WHO) World Health Organization; (ISBI) International Society for Burn Injuries; (GACC) Global Alliance for Clean Cookstoves; (CDC) Centers for Disease Control. Adapted using information provided from “The design and evaluation of a system for improved sun/eillance and prevention programmes in resource- limited settings using a hospital-based bum injury questionnaire” [14].

In response, the World Health Organization (WHO) developed the Global Burn Registry (GBR). The GBR is a system for voluntary hospital-based reporting of burn injury data. The GBR is a minimum dataset aligned with a centralized registry with the objective to collect key prevention, care and outcome data from burn injured patients in a standardized manner while minimizing the need for advanced information technology capacity [3]. The GBR also serves as a data sharing platform accessible to both contributing hospitals and the greater burn community for analysis that informs prevention initiatives and quality improvement programmes. It provides the much needed opportunity to transition from isolated and inconsistent approaches of recording burn injury data, to a standardized, comparable and consistent global data collection system [3]. See ***Figure 1*** for further details on the GBR.

Universal uptake of the GBR by facilities that care for burn injuries would help elucidate the true burden of these injuries and elevate the global discussion and approaches to burn injury prevention and care in a more contextually informed manner. However, since the introduction of the GBR, uptake and use by hospitals globally has been relatively low and inconsistent. Currently, data are not globally representative and selection bias, due to variable participation, significantly limits interpretation of epidemiological studies [4,5]. Other than internal WHO feasibility and pilot testing of GBR prior to its dissemination, there have been no studies on the implementation of the GBR. Therefore, to understand the key factors contributing to implementation, we conducted a survey aiming to identify barriers and facilitators to adoption and consistent use of the GBR.

## GBR Implementation Survey Development and Dissemination

**Figure 2:**
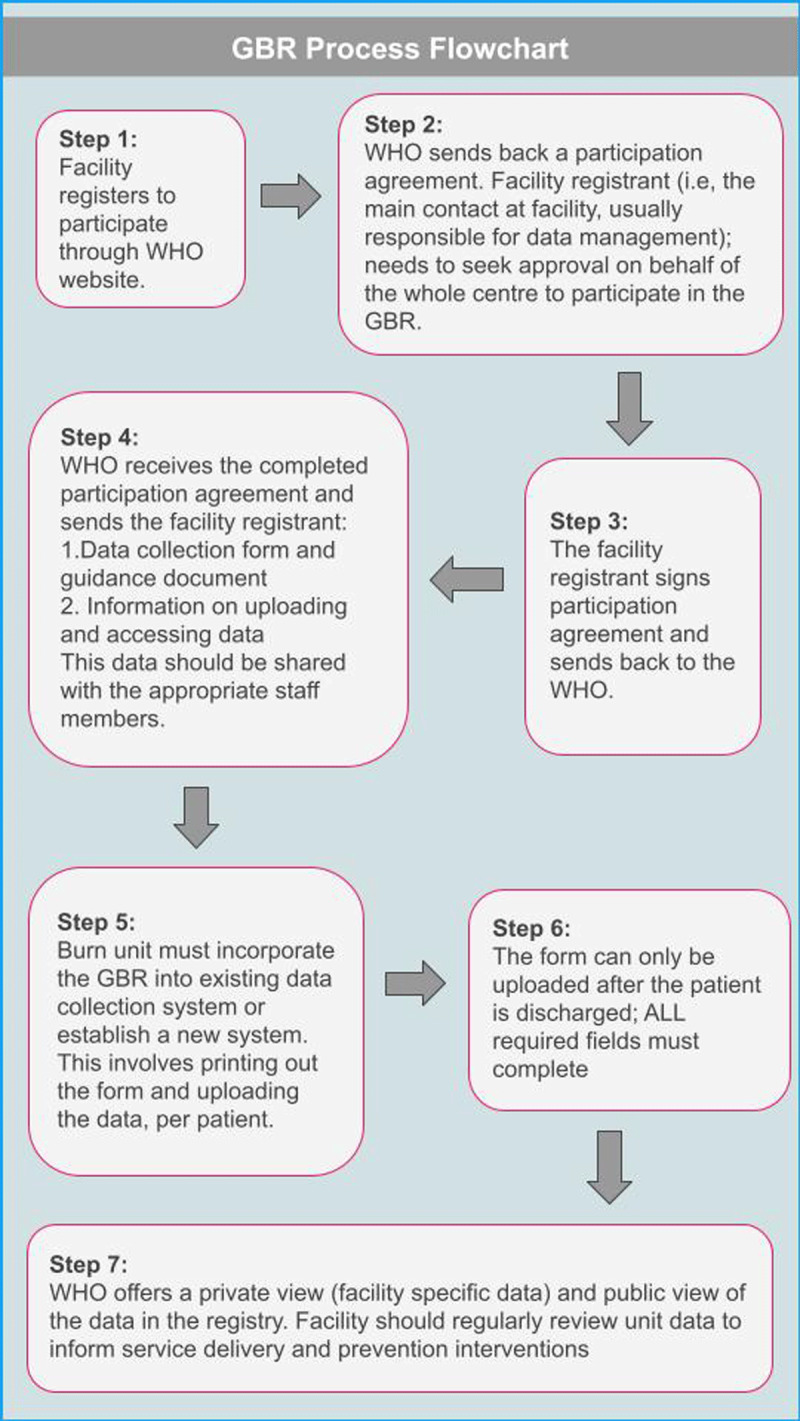
Global Burn Registry (GBR) user registration, set-up, and use process.

The Centre for Global Burn Injury Policy and Research at Swansea University created two separate implementation surveys designed to collect data from “GBR users” and “GBR non-users,” respectively. The surveys were reviewed by a WHO representative to ensure relevance and alignment with the GBR initiative. Survey items were included to capture burn facility information, details about participation in the GBR, and opened-ended questions regarding the utility of the GBR, GBR process and workflow, and lessons learned for GBR use and promulgation. The GBR non-user implementation survey included a thorough explanation of the GBR set-up and use process (***Figure 2***) and was accompanied by items that aimed to understand non-users’ perspectives on the anticipated barriers and facilitators to GBR adoption and use. The final surveys were developed as electronic forms on Qualtrics™ and were accessible via hyperlinks to the survey website.

We employed purposive sampling methodology. Facilities that had uploaded data onto the GBR were identified and contact details were obtained from the WHO (consent dependent). This mailing list was used to send the implementation survey for GBR users. The survey link for GBR non-users was shared via numerous electronic platforms through the international burn community (e.g., Interburns; International Society of Burn Injuries) and to burn centres where the GBR was not in use. The online surveys were completed on a voluntary basis by experts from respective GBR user and non-user facilities.

The primary objective of conducting the surveys was to inform the development of implementation support tools. Therefore, quantitative data were described and qualitative responses were analysed using a content analysis framework [6]. First, responses were coded by implementation major barriers and facilitators. Responses with similar implementation barriers and facilitators were coded and organized by more specific pitfall, process and/or solution. Facility-level and implementation process data were collected. No data on personal characteristics of any individuals were collected. Use of the data for this publication is considered not to constitute human subjects research by the Swansea University and University of Washington Institutional Review Boards.

## Survey Respondents

### GBR users

There were ten respondents from seven countries (***Table 1***). Two respondents were later found to be “GBR non-users” who completed the incorrect questionnaire. The majority of respondents reported that their facilities had a specialized burn unit (8 of 10 respondents) and had one or more dedicated burn surgeons (9 of 10 respondents). Most users also reported that more than 100 burn patients were admitted annually at their respective facility (9 of 10 respondents).

**Table 1:** Respondents to GBR implementation surveys.


	GBR USERS	GBR NON-USERS

**Number (n)**	10	33

Respondent Countries	*India, Mozambique, Nigeria, Peru, Tanzania, United Kingdom, Zambia*	*Australia, Brazil, Ethiopia, India, Israel, Mozambique, Sudan, United Kingdom, United States, Zambia*

**Burn patients admitted annually**		

<50	0	5

50–100	2	2

100–150	3	5

>150	5	11

**All burn patient data added to GBR? (Users only)**		

Yes	3	N/A

No	7	N/A

**Have you previously contributed to GBR (Non-users only)**		

Yes	N/A	6

No	N/A	27

**Any knowledge of GBR prior to survey? (Non-users, never contributed to GBR only)**		

Yes	N/A	11

No	N/A	15


### GBR non-users

Thirty-three respondents completed the GBR non-user survey and represented ten countries (***Table 1***). The majority of respondents reported that their facilities admitted more than 100 burn patients annually (16 of 21 respondents). Most non-users reported that their facility had never previously used the GBR (26 of 33 facilities); of those whose facilities had never used the GBR, less than half of the respondents had previously heard of the GBR prior to receiving the survey (11 of 26 respondents).

## Main Observations

### GBR users

Only 3 of 10 GBR users reported that data from all burn patients admitted to their facility were routinely uploaded into the GBR. At facilities that regularly contributed data, respondents stated that patient data were typically uploaded after each patient discharged or once per week (i.e., not prospectively or daily). From respondents whose facilities did not upload all patient data to the GBR, the most commonly cited barriers to consistent data collection and upload were lack of supervision or accountability, lack of standard operating procedures for data collection and upload within the facility, no dedicated personnel and/or limited protected time for participation (***Box 1***). Other commonly identified barriers for participation in the GBR were lack of support from senior managers, lack of interest from hospital staff and not having an identified a lead for the process.

**Box 1 Insights from GBR users**

- Frequent and routine data uploads (e.g., per patient or weekly) may facilitate consistent and complete data entry into the registry.
- A strong emphasis by facility leadership regarding the mission and utility of the GBR is essential to maintaining motivation and consistency in participation.
- Dedicated personnel and standard operating procedures improve participation.

### GBR non-users

When presented with a demonstration of the steps required for GBR participation (***Figure 2***), the most commonly anticipated barrier steps (e.g., steps perceived as challenging to complete) were: 1) setting up GBR process (e.g., receiving, training, setting up the system for data collection), and 2) regularly using and reviewing facility-level data for prevention and quality improvement purposes. Slightly more than half of the respondents who had never contributed to the GBR felt it would be a useful tool for data sharing and comparison, advising operations/logistics of burn care, benchmarking quality improvement programmes, and informing prevention initiatives and policy. However, nearly half of the respondents felt that the GBR would not be useful or were unsure of the utility of participating in the GBR. The reasons cited included participation in other registry programmes (e.g., hospital- or national-level), GBR dependence on paper data collection forms (i.e., not phone or tablet-based software), and perception that the low levels of current participation in the GBR hinder its potential for large-scale impact (***Box 2***).

**Box 2 Insights from GBR non-users**

- Setting-up the GBR and related data management processes is a major perceived barrier for many non-users.
- Many stakeholders in the burn care community are unaware of the GBR.
- There is uncertainty around the utility of the GBR, indicating a need for demonstrable benefits, education, and promotion to maximize global participation.

### Identified process pitfalls and best practice solutions from GBR users and non-users

***Table 2*** demonstrates some specific pitfalls experienced by respondents and offers best practice solutions to overcome them, as informed by identified barriers and facilitators.

**Table 2:** Process pitfalls and best practice solutions described by Global Burn Registry (GBR) users and non-users.


IDENTIFIED PROCESS PITFALL	PROPOSED BEST PRACTICE SOLUTIONS

GBR sign-up andset-up process.	– Utilize available resources for GBR use and implementation such as Interburns Global Burn Registry online module: – Using the GlobalBurn Registry (GBR) – Overview|Rise 360 (articulate.com) – Consider contacting a peer facility with experience in GBR use for consultation.

Obtaining buy-in and engagement from management and/or supervisors.	– Demonstrate the crucial role of standardized data collection for quality improvement programmes at participating facilities. – Look to obtain additionally support and external advocacy from Ministry of Health, regional WHO (e.g. PAHO, etc.) or WHO office.

Establishing and/or sustaining staff motivation.	– Emphasize utility of the GBR to inform injury control and prevention – Conduct regular “check-in” meetings to educate and motivate staff, as well as celebrate small milestones in the process

Inconsistent data collection.	– Develop a system for data collection: Appoint roles and responsibilities for who collects specific portions of information. For example, the physician may fill the initial assessment and the nurse completes the form at discharge. Have senior personnel who oversees compliance, completeness, and quality control of the paper forms.

Inconsistent data uploads.	– Develop a system for data transcription and upload: If there are pre-existing data management personnel, they should be assigned the task of uploading data from the paper form onto the electronic database. If this role does not exist, keep all the paper forms together in a predetermined place and have a rotating system for staff to transcribe and upload the data, either per patient or once a week, to share the workload associated with uploading. Have senior personnel who supervise upload process.

Inconsistent internet connection.	– Develop a system of intermittent uploads that aligns with times of greater internet bandwidth (e.g. plan for data uploads at night).

Misplacing/difficulty tracking paper form.	– Print data collection form on brightly coloured paper. –Have involved staff develop an accepted filing system for the GBR.

Utilizing GBR data for quality improvement.	– Participating facilities can develop a plan to review the GBR inputs regularly (i.e. once per month, or more frequently). –Develop working groups in a virtual *community of practice*.


## Lessons Learned

The WHO GBR has the potential to be a very powerful tool for collating facility-, national- and global-level burn injury and care data to describe epidemiology and inform prevention and quality improvement initiatives. The implementation surveys shed light on some of the barriers and facilitators to adoption and use of the GBR. The main themes of lessons learned are: 1) coordinate education and promotion, 2) develop an implementation toolkit for facility-based quality improvement, and 3) create a community of practice. It is essential to utilize this information to support current and future stakeholders and promote the use of the GBR. Given the voluntary nature of survey participation, the lessons learned may not represent all user experiences; however, the key learning points from stakeholders’ experiences can still inform steps to enhance engagement with the GBR. By doing so, we may realize further progress for burn injury prevention and care globally. Further, these lessons may also be broadly applicable to those implementing registries globally, regardless of specialty focus.

### Educate stakeholders and promote the GBR

Numerous stakeholders within the burn community have little to no exposure to the GBR or its utility. Thus, we recommend coordinated education efforts, marketing, and promotion of the GBR by the WHO and relevant professional societies to demonstrate the utility of the registry. One approach to do so might include highlighting the programmatic uses of the GBR. For example, WHO could compile and publish GBR success stories to showcase the potential of stakeholder investments in and experiences utilizing the GBR. Similar work has been done to support WHO trauma and injury prevention-related products, like the *WHO Strengthening Care for the Injured: Success Stories and Lessons Learned from Around the World* [7]. The case studies within this compilation were found to be broadly utilized and demonstrated the power of disseminating lessons learned [8]. Another potential strategy could include collaborating with professional societies (e.g., International Society for Burn Injuries, International Association for Trauma Surgery and Intensive Care) to conduct GBR informational and training sessions through webinars or during annual international meetings. Additionally, society journals might consider publishing “GBR pearls,” similar to the methods the American Burn Association employed during both early and current iterations of the National Burn Repository [9,10].

### Develop an implementation toolkit for quality improvement

The stakeholders recommended developing a GBR implementation toolkit to kickstart and maintain data collection and entry as well as facilitate quality improvement (QI) programming. It has been demonstrated that access to real-time data feedback can yield rapid, significant and sustained improvement in the quality of care as well as elevate staff motivation and job experience, including at hospitals without formal QI programmes in LMICs [11,12]. Further, successful QI programming can improve outcomes like complication rates and length of stay, which can directly decrease hospital expenditures. Therefore, by demonstrating improved quality of care and costs-saving potential of using GBR, administrators and service-line directors may be more willing to support the necessary staffing, equipment, and resource requirements for effective registry workflow.

As example of how support for QI can be enhanced, the *WHO International Registry for Trauma and Emergency Care (IRTEC)*, a web-based platform, minimum dataset and analysis system for case-based data from emergency care and injury encounters, provides a number of accessible automated reports to facilitate QI programming [13]. Given the existing WHO technology platforms, a similar automated analysis and reporting system would be a useful next step to expand the functionality of the GBR and can be paired with user guides as part of the implementation toolkit.

### Create a community of practice

The surveys demonstrated similar experiences found by the post-pilot questionnaire, that limitations in staff motivation, training, administrative support and office supplies challenge consistent data collection and entry [14]. While this may not address every issue encountered, we recommend developing a system of continuous process improvement for GBR users by establishing an organized community of practice among GBR stakeholders. Members of the community can share their experiences of implementation, successes, troubleshooting, and process management techniques (e.g., achieving management buy-in, staffing solutions, resource optimization). This group can participate asynchronously through virtual and instant messaging platforms, and/or at bi-annual virtual conferences organized for stakeholders. Virtual communities of practice–including multidisciplinary communities—have been utilized as a means of support and professional development in various clinical and public health disciplines to bolster specific interventions [15,16,17].

### Perform periodic evaluations

The promulgation and functions of the registry should be routinely evaluated. To our knowledge, metrics of success and goals for the GBR have not been publicly defined or published. We suggest establishing key metrics and indicators and measure them at set intervals. Such measures might include number of contributing facilities, country/regional representation, completeness of data, rate of facilities joining, number of publications, and instances of utilization to inform burn injury prevention and control initiatives. A similar evaluation via systematic review was conducted to understand the dissemination and uptake of the *WHO Guidelines for Essential Trauma Care* and presents another option for evaluation of GBR use [18]. Serial surveys can be sent to GBR users to identify how the GBR has impacted patient care and outcomes, user “best practices” (which could be used as “GBR Pearls” for education and promotion) and opportunities for iterative adjustment and evolution of the GBR to fit stakeholder needs. For example, the desire for a mobile device-enabled data entry application option (in addition to the current paper data form/transcription process)—as was recommended by some stakeholders in this survey—could be re-assessed and trialled in the future, and potentially expand the functionality of and participation in the GBR.
